# Dexrazoxane ameliorates radiation-induced heart disease in a rat model

**DOI:** 10.18632/aging.202332

**Published:** 2021-01-02

**Authors:** Long Li, Xiaoqi Nie, Peng Zhang, Yongbiao Huang, Li Ma, Fang Li, Minxiao Yi, Wan Qin, Xianglin Yuan

**Affiliations:** 1 Department of Oncology, Tongji Hospital, Huazhong University of Science and Technology, Wuhan, Hubei Province, China

**Keywords:** radiation-induced heart disease, dexrazoxane, apoptosis, reactive oxygen species, rat model

## Abstract

Treatment of thoracic tumors with radiotherapy can lead to severe cardiac injury. We investigated the effects of dexrazoxane, a USFDA-approved cardioprotective drug administered with chemotherapy, on radiation-induced heart disease (RIHD) in a rat model. Male Sprague-Dawley rats were irradiated with a single dose of 20 Gy to the heart and treated with dexrazoxane at the time of irradiation and for 12 subsequent weeks. Dexrazoxane suppressed radiation-induced myocardial apoptosis and significantly reversed changes in serum cardiac troponin I levels and histopathological characteristics six months post-radiation. Treatment with dexrazoxane did not alter the radiosensitivity of thoracic tumors in a tumor formation experiment using male nude Balb/C mice with tumors generated by H292 cells. Dexrazoxane reduced the accumulation of reactive oxygen species in rat cardiac tissues, but not in tumors in nude mice. Transcriptome sequencing showed that *IKBKE, MAP3K8, NFKBIA,* and *TLR5*, which are involved in Toll-like receptor signaling, may be associated with the anti-RIHD effects of dexrazoxane. Immunohistochemistry revealed that dexrazoxane significantly decreased NF-κB p65 expression in cardiomyocytes. These findings suggest dexrazoxane may protect against RIHD by suppressing apoptosis and oxidative stress in cardiomyocytes.

## INTRODUCTION

Approximately 70% of all cancer patients receive radiotherapy at some point during their treatment [1]. Oncologists are actively examining the adverse effects of radiotherapy on the heart. The first case of radiotherapy-induced cardiac death was reported in 1963 [2]. In addition, increased rates of RIHD have been observed in long-term survivors of breast cancer [3] and lymphoma [4] over decades of follow-up. Moreover, RIHD is detected shortly after the completion of radiotherapy in patients with some types of tumors. Approximately 10.3% of patients with locally advanced lung cancer experience major adverse cardiac events (MACE) within 20 months after radiotherapy. The risk of MACE was significantly higher in patients who received a mean heart dose (MHD) of more than 10 Gy [5]. Furthermore, the risk of major coronary events increased linearly with MHD by 7.4% per Gy and persisted up to 30 years after radiotherapy in breast cancer patients [3]. Although cardiac damage caused by irradiation persists for long periods, relatively little is known about this condition. A Norwegian study on long-term survivors of childhood malignant lymphomas reports that, although 34% of patients were aware of the risk of long-term side-effects of radiotherapy, only 13% routinely attended follow-ups [6].

Cardiovascular toxicity is the leading cause of death in long-term survivors of breast cancer, especially in patients with early stages [7], and the second leading cause of mortality in cancer survivors [8]. The number of cancer survivors globally is expected to pass 22 million by 2030 due to advances in medical treatment and increases in lifespan in general [9]. As the number of cancer survivors increases, the number experiencing RIHD would also likely increase, possibly offsetting some benefits of thoracic radiation. Epidemiological studies have improved our understanding of RIHD, and increased precision in radiation dosing techniques results in smaller doses being absorbed by the cardiovascular system. However, there are no clinically effective drugs that can be used to prevent or reverse the onset and progression of RIHD [10].

The growing field of “cardio-oncology” is examining the cardiotoxic effects of some anti-tumor drugs, including anthracyclines, antimetabolites, and targeted drugs [11–13]. Cardiotoxicity induced by antineoplastic drugs is partly associated with oxidative stress (OS) [14]. Excessive accumulation of free radicals and reactive oxygen species (ROS) can damage mitochondrial function, cause cell damage, trigger cell apoptosis, and consequently lead to cardiac dysfunction [15]. Although the exact molecular mechanisms of RIHD are not completely understood, endothelial and mitochondrial injuries, the endoplasmic reticulum, and OS play significant roles in its etiology. Radiation damage to normal tissues is due to direct exposure to high intensity radiation energy and indirect oxidative stress caused by ROS. Because of its weak antioxidant defense, the myocardium is susceptible to oxidative damage caused by free radicals generated during irradiation [16].

Pharmacological agents including beta blockers, statins, and dexrazoxane (DZR) demonstrate potential cardioprotective effects in patients receiving anthracycline or trastuzumab, although more extensive clinical trials are needed to evaluate these effects [13]. DZR is recommended by the USFDA to prevent anthracycline cardiotoxicity. DZR significantly reduces anthracycline-induced cardiotoxicity without altering its antitumor efficacy, dosing frequency, toxicity, or secondary malignancy rates compared to control groups [17–20]. However, the role of DZR in RIHD has not yet been studied to our knowledge.

In this study, we examined whether DZR played a cardioprotective role and prevented radiation-induced damage in a rat model of RIHD.

## RESULTS

### DZR ameliorates myocardial injury induced by irradiation in rats

**Figure 1:**
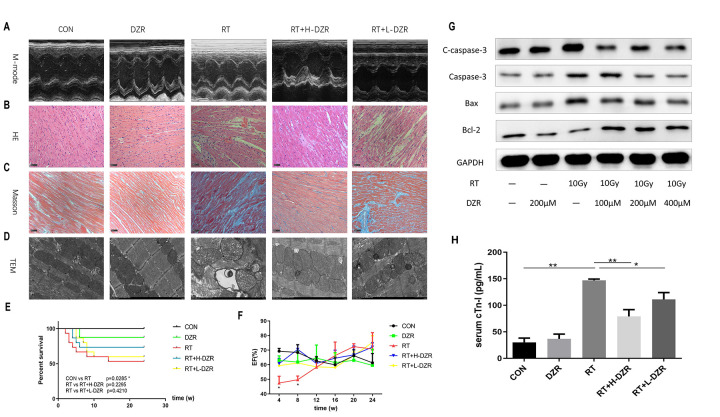
**Dexrazoxane decreases functional damage and structural injury in the rat heart after irradiation.** (**A**) Representative M-mode echocardiograms for each group. (**B**, **C**) Representative HE and Masson staining (×200, scale bar =100 μm) of hearts from each group (n=7-11, respectively). (**D**) Subcellular abnormalities in mitochondria, sarcomeres, and myofilaments identified by TEM (×10K). (**E**) Kaplan-Meier analysis of rat survival in each group. Exposure to whole heart irradiation significantly reduced overall survival time (CON vs RT, *p* =0.029). A non-significant trend for a long-term survival benefit of DZR was observed. (**F**) Irradiation significantly decreased EF in rats within 8 weeks. No significant differences were observed among the groups (n=3 at every time point for each group) after 8 weeks. (**G**) H9C2 cells were pre-treated with or without DZR (100, 200, and 400 μM) before a single 10 Gy X-ray irradiation. 24 h after irradiation, radiation-induced apoptosis-related injuries were reduced in cardiomyocytes pre-treated with 200 and 400 μM DZR. (**H**) Serum levels of cardiac troponin I (cTnI) were assessed at week 24 after irradiation. cTnI levels in CON and DZR groups were 30.13 ± 7.94 and 36.58 ± 8.97 pg/mL (n=5), respectively. Irradiation significantly increased cTnI levels in irradiated rats. cTnI levels were as follows: 147.00 ± 2.46 pg/mL in RT group (vs CON, p<0.01, n=8), 78.95 ± 12.81 pg/mL in RT+ H-DZR group (vs RT, *p*<0.01, n=11), 110.80 ± 11.36 pg/mL in RT +L-DZR group (vs RT, *p*<0.05, n=9). Data are expressed as mean ± SEM, *: *p* <0.05, **: *p* <0.01.

To investigate the radioprotective effect of DZR in cardiomyocytes, we examined changes in the levels of proteins related to the apoptotic pathway in H9C2 cells after radiotherapy. Different concentrations of DZR (100 μM, 200 μM, and 400 μM) were added to H9C2 cells an hour before they received 10 Gy irradiation. The effects of DZR on radiation-induced myocardial cell injury were assessed 24 hours later by a Western blot apoptosis assay. As shown in Figure 1G, expression of the proapoptotic proteins Bax, Caspase-3, and cleaved caspase-3 (C-caspase-3) increased, while expression of the antiapoptotic protein Bcl-2 declined markedly, in irradiated H9C2 cells. Furthermore, DZR pretreatment (200 or 400 μM) inhibited the radiation-induced increase in pro-apoptotic protein expression and decrease in Bcl-2 expression. These results indicate that the radioprotective effects of DZR *in* *vitro* were partially due to an inhibition of pro-apoptosis signaling.

Next, we used a rat model to evaluate the cardioprotective role of DZR in radiation-induced heart injury *in vivo.* Echocardiography was performed every 4 weeks to assess changes in cardiac function associated with RIHD. Type M-mode sonograms used to measure left ventricular ejection fraction (EF) are shown for each group in Figure 1A. EF decreased significantly in RT rats compared to CON rats at 4 (*p*=0.012) and 8 weeks (*p*=0.034). This reduction was significantly reversed in the RT+ H-DZR treatment group at 8 weeks (*p*=0.007), but no significant differences were observed between the groups during the subsequent observation period, suggesting the presence of an acute injury with a later recovery (Figure 1F). Histological evaluation also showed that DZR protected against RIHD as indicated by decreases in chronic fibrosis. HE staining (Figure 1B) and Masson’s trichrome staining (Figure 1C) revealed that cardiomyocytes in RT rats exhibited breakdown, edema, disordered arrangement, twisted myocardial fibers, nuclear condensation, interstitial fiber deposits, and increased numbers of inflammatory cells. Furthermore, transmission electron microscopy (TEM) showed evidence of profound cardiomyopathy-associated subcellular changes. Mitochondria of RT rats exhibited hyperplasia and hypertrophy, appearing swollen with widened or broken cristae. In addition, abnormal sarcomeres, expanded sarcoplasmic reticulum, myofilament fractures, partial focal dissolution, widening of the myo-interstitial space, and deposition of collagen fibers were observed in RT rats. These abnormalities were partially alleviated by DZR treatment, especially in the high dose group (Figure 1D). At week 24, 7 rats in the RT group had died (most within 8 weeks after irradiation), while 4 and 6 rats had died in the RT+ H-DZR and RT+ L-DZR groups, respectively. Although DZR treatment appeared to prolong survival in rats, this effect did not reach statistical significance (Figure 1E). Serum cardiac troponin I (cTnI) levels were significantly increased in the RT group compared to the other groups, suggesting that myocardial injury occurred in rats exposed to radiation. However, this increase was reduced in the RT+ L-DZR (*p*=0.02) and RT+H-DZR (*p*<0.01) groups (Figure 1H). These results indicate that the rat model of RIHD was successfully established and that DZR may protect against the development of RIHD.

### Treatment with DZR does not alter radiosensitivity of thoracic cancer *in* *vivo*

**Figure 2:**
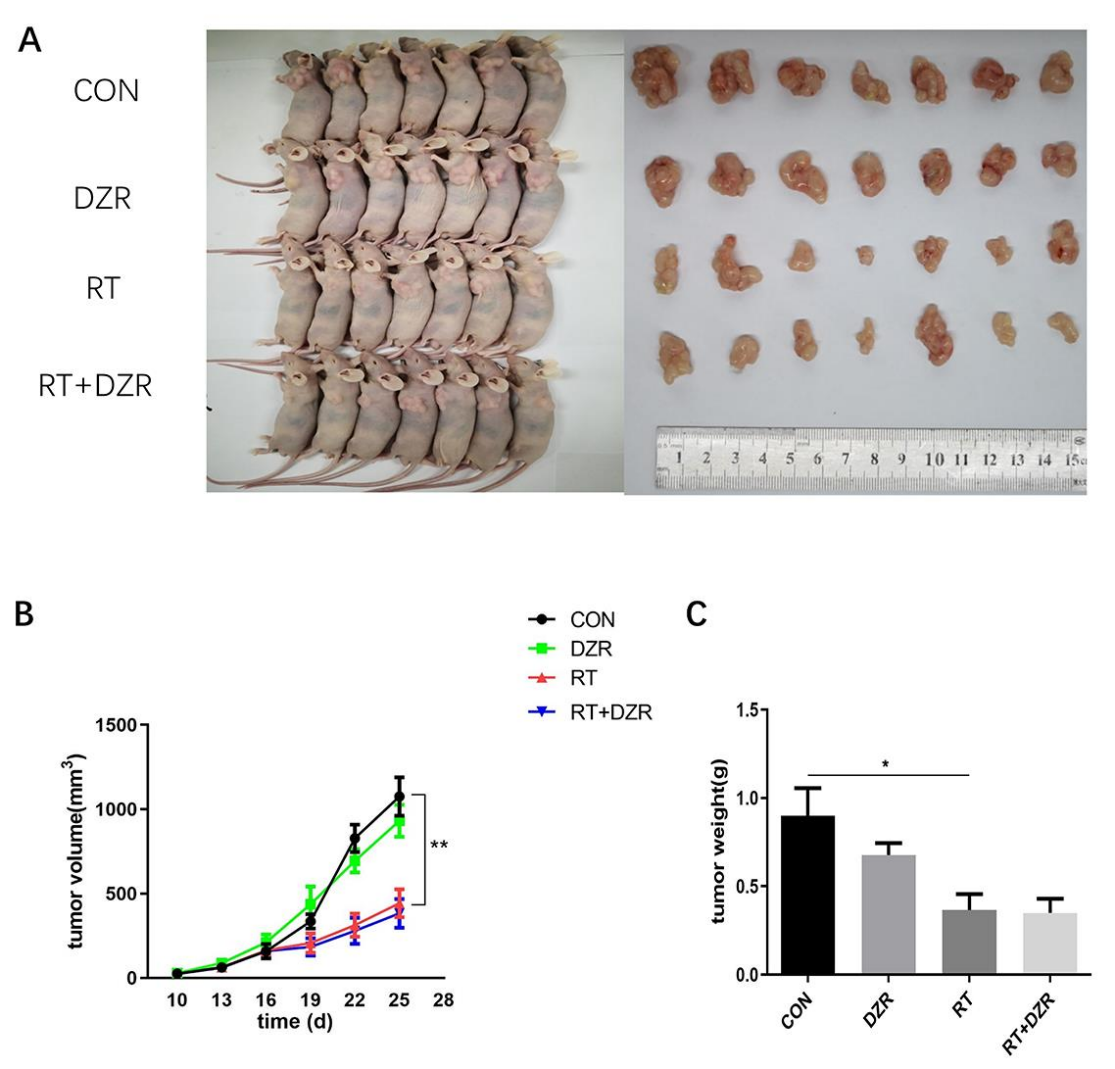
**Radiotherapy combined with DZR treatment does not inhibit tumor growth *in vivo*.** (**A**) Tumor images for each group. (**B**) Tumor volume changes in nude mice over time. (**C**) Final tumor weight in nude mice; data are expressed as mean ± SEM, *: *p* <0.05, **: *p* <0.01**.**

After establishing the radioprotective effects of DZR on RIHD, we used a xenograft model to investigate whether DZR reduced radiosensitivity in thoracic tumors *in vivo*. In a subcutaneous tumor model generated by transplanting H292 cells, tumor volumes met the criteria for establishing experimental groups at 10 days after injection. There were no significant differences in tumor volume among the four groups during the initial observation period. Irradiation significantly decreased tumor volume and weight compared to the control groups beginning at 12 days after treatment (*p*<0.01), but no obvious differences were observed between the RT group and the RT+DZR group. (Figure 2A–2C). These results indicated that DZR did not reduce the sensitivity of thoracic tumors to radiotherapy in nude mice.

### DZR inhibits RIHD-associated apoptosis by decreasing ROS generation in heart tissue, but not in tumor tissue, after irradiation

**Figure 3:**
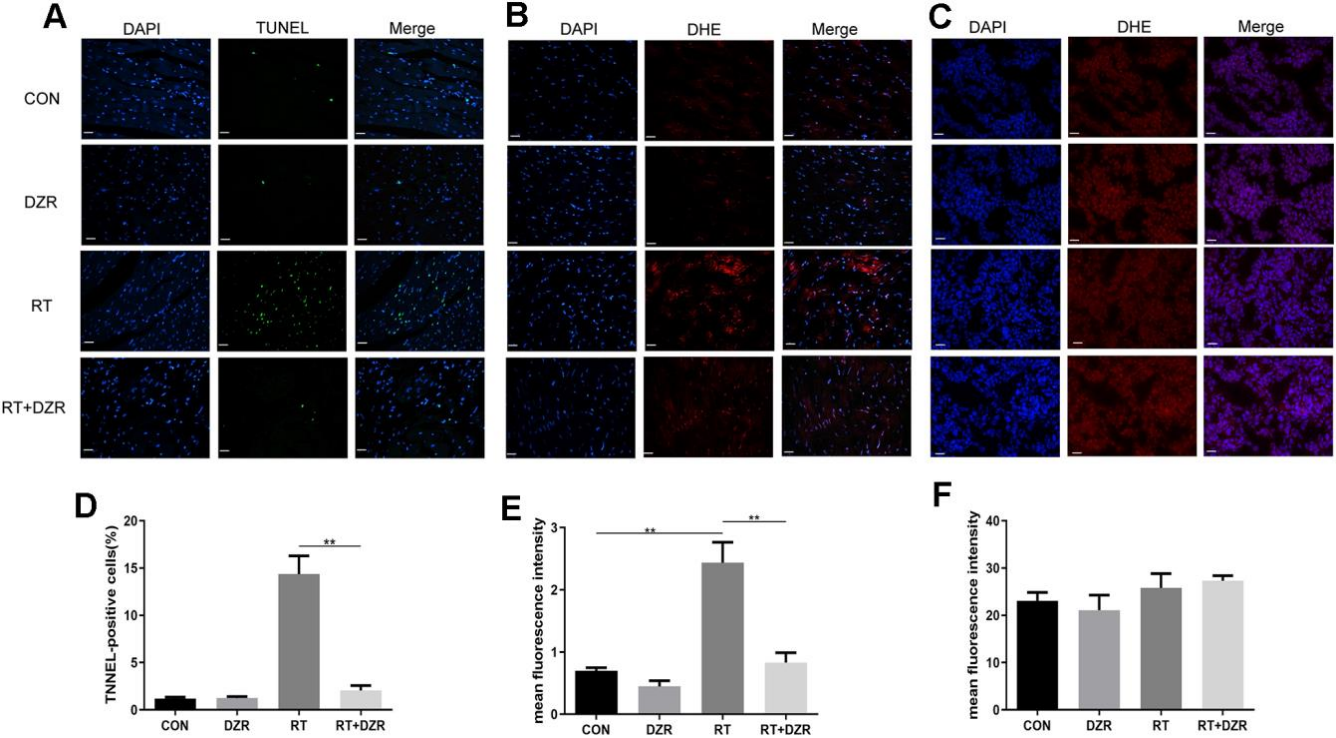
**Effect of DZR on post-radiation ROS generation in heart and tumor tissue**. (**A**, **D**) show typical TUNEL-stained photomicrographs of heart tissues (×400, n=7–11 respectively). TUNEL- and DAPI-positive cells appear green and blue, respectively. Myocardial cell apoptosis increased markedly in RT rats compared to other groups. (**B**) Representative images of dihydroethidium (DHE) fluorescence staining (×400) of rat cardiomyocytes. DHE=red, nuclei=blue. (**E**) Quantitative analysis of ROS (n=5 per group) in rat heart tissues. (**C**) Representative microphotographs of DHE staining (×400) in tumor tissues from nude mice. (**F**) No differences in DHE fluorescence density of tumor tissues were observed in quantitative analysis (n=5 per group). mean ± SEM, *: *p* <0.05, **: *p* <0.01. scale bar =50 μm.

Numbers of apoptotic cardiomyocytes were significantly higher in RT group rats than in CON and RT+H-DZR group rats (referred to as RT+ DZR) for 6 months after irradiation (Figure 3A). The apoptosis rate was 14.37 ± 1.92% in the RT group and 2.04 ± 0.54% in RT+ DZR group (*p*<0.01) (Figure 3D). This suggests that DZR reduced radiation-induced apoptosis in cardiomyocytes *in vivo*. In order to investigate the antioxidant activities of DZR, we used a DCFH-DA fluorescence assay to measure ROS in frozen cardiomyocytes from rats and in tumors from nude mice. We found that irradiation increased ROS generation in the rat heart compared to the CON group (*p*<0.01) (Figure 3B, 3E). Furthermore, combined treatment with DZR significantly reversed radiation-induced ROS generation (*p*<0.01). However, no obvious fluorescence intensity differences indicative of ROS production were observed in tumors from nude mice among the experimental groups (Figure 3C, 3F). This suggests that DZR reduced apoptosis in normal heart tissues without altering the therapeutic effects of radiotherapy. This might be a result of selective suppression of radiation-induced ROS generation in rat cardiomyocytes and not in tumor tissue from nude mice.

### Reduction of Toll-like receptor/NF-κB signaling pathway mRNA expression is involved in the anti-RIHD effects of DZR in the rat model

**Figure 4:**
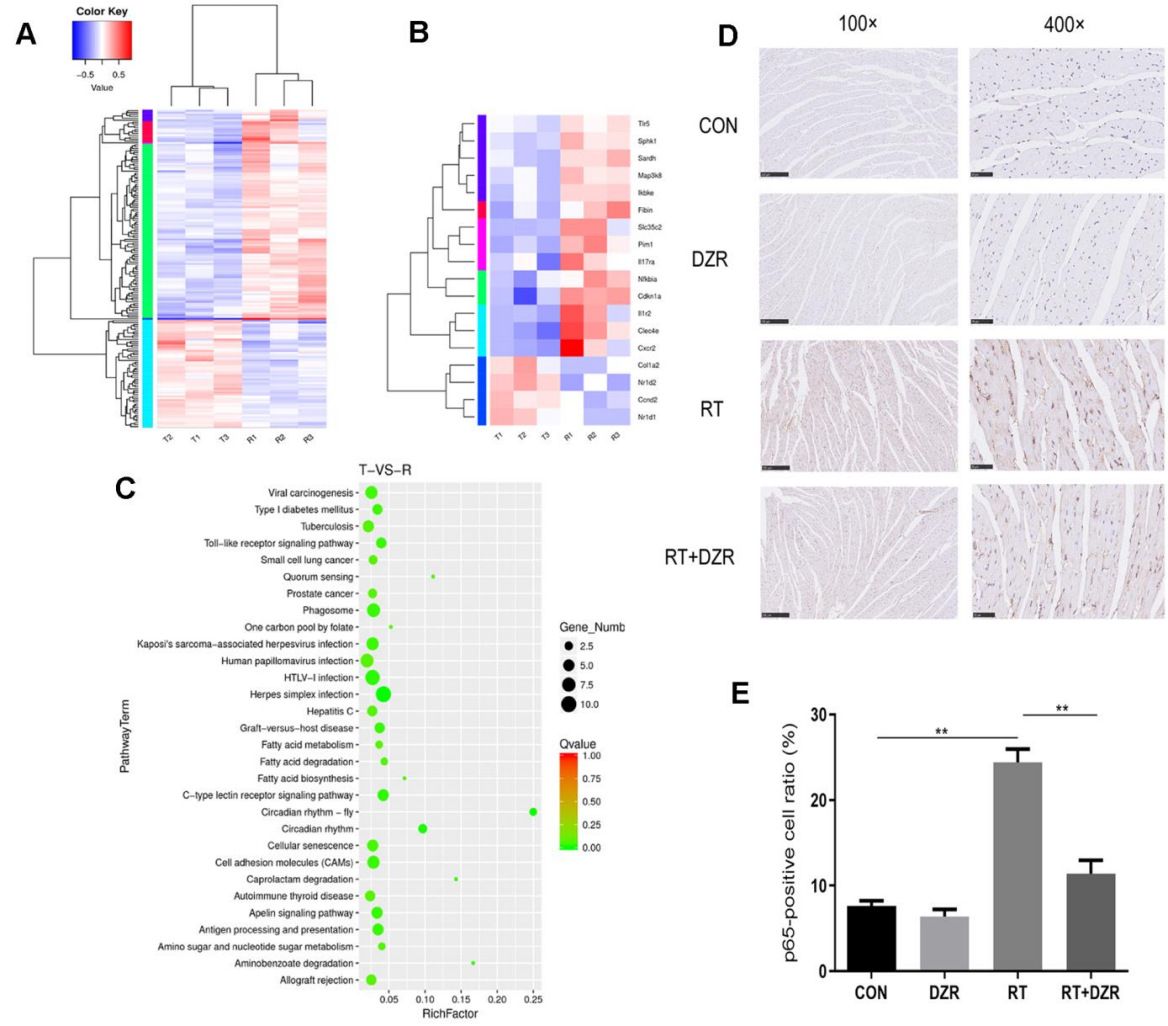
**Gene expression profile in heart tissue from the RIHD rat model.** (**A**) Results of transcriptome sequencing of tissue samples from RT and RT+H-DZR rats. (**B**) Gene expression profiles of Toll-like signaling pathway members were examined using a heat map. Red, high expression; white, intermediate expression; blue, low expression. (**C**) Top 30 enriched pathways identified using distribution points of differential genes in KEGG pathway analysis. The horizontal axis represents the enrichment factor, point size indicates the number of differential genes in the pathway, and the color of the points corresponds to different Q-value ranges. T indicates RT+H-DZR, R indicates RT. (**D**, **E**) show NF-κB-p65 protein levels (×100, scale bar =250 μm; ×400, scale bar =50 μm) in myocardial tissue determined by immunohistochemistry (n=5 per group). mean ± SEM, *: *p* <0.05, **: *p* <0.01.

In order to elucidate the mechanisms underlying the protective effects of DZR against RIHD, gene expression in cardiac tissue from RT+ DZR and RT group rats was analyzed using transcriptome sequencing (Figure 4A). The heat map revealed that transcription of four genes involved in the Toll-like receptor signaling pathway, *IKBKE, MAP3K8, NFKBIA,* and *TLR5*, was significantly inhibited in the RT+ DZR group compared to the RIHD group (Figure 4B). KEGG pathway analysis of gene set enrichment indicated that gene clusters associated with the Toll-like signaling pathway were downregulated after DZR treatment (Figure 4C). Immunohistochemical analysis revealed that NF**-**κB subunit p65 protein expression was significantly higher in cardiomyocytes from the RT group (24.42%) compared to the control group (7.61%) (*p*<0.01). Furthermore, p65 expression was significantly decreased in RT+ DZR rats (*p*<0.01, Figure 4D, 4E). These findings suggest that the Toll-like/NF-κB pathway was important for the anti-RIHD effects of DZR; this pathway might therefore also play an important role as a downstream effector after activation by ROS.

## DISCUSSION

Advances in precision radiotherapy techniques can be accompanied by adverse effects which extend to several organs. Thoracic radiation therapy can potentially affect all cardiac structures. Patients may develop RIHD, which decreases the survival benefits of anti-cancer treatment, several years after treatment has ended [21]. Strategies to protect against RIHD are therefore needed. A multicenter phase III clinical trial showed that DZR significantly alleviates anthracycline-induced cardiac events (13% vs 39%, p<0.001) [22]. The European Society of Cardiology states that DZR significantly reduces the risk of heart failure in adult cancer patients treated with anthracycline without affecting the tumor-response rate [23]. In this study, we demonstrated the cardioprotective effects of DZR both *in vitro* and *in vivo*.

Among stage III NSCLC patients treated with chemoradiotherapy, overall survival was higher among those treated with 60 Gy radiation than those treated with 74 Gy, and excessive cardiac irradiation doses were a predictor of poor survival. Incidence of RIHD significantly increased when the mean dose to the heart was more than 20 Gy [24–26]. Relatively few animal studies examining RIHD have been conducted, and most are based on rat models. One study found that when the dose of a single radiation administered to the rat heart increased from 18 Gy to 24 Gy, cardiomyocytes were more severely damaged or were even replaced by interstitial fibrosis within six months after irradiation [27]. In the present study, partial myocardial fracture and interstitial fibrosis were observed in histological assessments of the rat heart after a single irradiation of 20 Gy, which was consistent with previous studies [28]. In addition, most of the rats that died after radiotherapy did so within eight weeks, which is consistent with echocardiography results showing significant EF reduction within eight weeks after radiotherapy. This suggests that acute cardiac injury after radiotherapy might have been the main cause of death in the animals in this study. Although we did not observe a significant DZR-associated improvement in rat survival times after irradiation, acute radiation damage to numerous other vital organs around the heart, such as the lungs, thymus, and esophagus, might also have caused the deaths of experimental animals in this study after high doses of radiation. We therefore evaluated the radioprotective effects of DZR on the heart in histopathological examinations.

DZR markedly inhibited the expression of pro-apoptotic proteins in H9C2 cells and reduced the number of apoptotic cardiomyocytes in rats after exposure to radiation. Apoptosis is an important pathological mechanism in radiation-induced tissue damage and is also observed in radiation-induced pulmonary fibrosis [29]. RIHD develops many years after irradiation in a dose-dependent manner, and the damage worsens over time, leading to fibrosis in heart tissue [30]. Apoptosis rate is closely associated with the severity of radiation-induced myocardial injury [31]. In the present study, histological evaluation showed myocardial interstitial fibrosis and abnormalities in cardiac mitochondria six months after radiation. The pathological developments in our rat model were therefore consistent with those observed in RIHD. In addition, mitochondrial damage was observed when myocardial tissues from irradiated rats were examined using TEM. Mitochondria are particularly susceptible to radiation-induced damage, and mitochondrial damage is related to changes in levels of apoptosis proteins including Bax, which are associated with the severity of radiation-induced cardiac injury [16]. Cardiac troponins are also markers of myocardial injury. In a prospective clinical study, serum troponin was an effective predictor of chemotherapy-related cardiac injury, suggesting that troponin-targeting treatment strategies may be effective [32]. Serum cTnI levels increased significantly after radiation and improved after DZR treatment in our study. However, echocardiography did not show similar results. We observed a decrease in EF soon after radiation, but this decrease disappeared over time. In contrast, a significant radiation-induced increase in EF was observed in another rat RIHD model after fractionated doses of 9 Gy for 5 fractions [33]. Our results are consistent with those of another previous study which found no differences in EF between the radiated and control groups in a rat RIHD model 5 months post-radiation when a single high dose of 20 Gy was used [34]. It is possible that a decrease in EF after radiation might be observed only after higher radiation doses and prolonged follow-up. Additionally, changes in EF might not always accompany myocardial injury, apoptosis, and interstitial fiber deposits, perhaps due to compensatory mechanisms in the heart. In summary, our results suggest that DZR may protect against RIHD.

Although RIHD is not well-understood, it is associated with an imbalance between the production and scavenging of ROS, which results in damage to cellular lipids, proteins, and DNA [35]. The heart is particularly vulnerable to ROS damage because it has lower antioxidant levels than other tissues and because its membrane structures are rich in phospholipids that are particularly sensitive to ROS [36]. Studies demonstrate that both acute and chronic excessive ROS production caused by antineoplastic drugs promote pathogenesis in cardiovascular disease [37]. Here, we found that irradiation caused mitochondrial damage and increased ROS production in the heart. This indicates that the anti-RIHD effects of DZR may be related to reduced ROS levels in the heart. At the same time, irradiation can damage the mitochondria and induce apoptosis of both cardiomyocytes and tumor cells. However, the Warburg effect in tumor cells enables a switch from aerobic oxidation to glycolysis in mitochondria; this metabolic change does not occur in cardiomyocytes [38]. Moreover, abnormally high ROS levels in tumor cells do not cause significant increases in tumor cell death, and malignant tumor development is itself associated with abnormal accumulation of ROS [39]. Given the complexity of oxidative stress regulation in tumor cells, we directly investigated whether DZR affects the efficacy of radiotherapy *in* *vivo*. Tumor formation experiments in nude mice indicated that DZR had no significant effect on radiotherapy efficacy in tumor tissues. This might be due to the large amount of ROS continuously produced during tumor-cell proliferation or to differences in ROS regulation mechanisms between cardiomyocytes and tumors. Transcriptome sequencing revealed that genes in the Toll-like signaling pathway were responsible for the therapeutic effect of DZR, and quantification of NF-κB p65 by immunohistochemistry in cardiac tissues confirmed that finding. Upon stimulation by activating inhibitor protein IκB, NF-κB regulates several pathophysiological processes, including inflammation and apoptosis. ROS also impact the NF-κB signaling pathway as a second messenger signal [40], possibly explaining the role of ROS in RIHD.

Some limitations of the present study should be considered when interpreting the results. First, we did not observe a significant improvement in EF or survival after DZR treatment, which may be related to the radiation dose used. A higher radiation dose should therefore be considered in future studies. Second, we did not include other potentially effective drugs as controls when evaluating the cardioprotective effects of DZR on RIHD. Lastly, the relationship between ROS, apoptosis, and NF-κB needs to be studied further to fully elucidate the mechanism underlying DZR’s anti-RIHD effects.

In conclusion, DZR treatment both before and after radiation significantly improved RIHD in a rat model without reducing the therapeutic effect of radiotherapy in a tumor formation experiment. The mechanisms underlying this effect might be related to OS and should be further examined in future studies.

## MATERIALS AND METHODS

### Cell cultures and irradiation

H292 cells were obtained from Cell Bank, Chinese Academy of Sciences, China and maintained in RPMI/1640 supplemented with 10% fetal bovine serum (Gibco, USA), penicillin (100 U/mL), and streptomycin (100 Ag/mL) with 5% CO_2_ at 37° C. Cells were irradiated with a single dose of 8 or 10 Gy using a clinical linear accelerator (6Mv X-rays, Elekta Precise, Stockholm, Sweden). In *in vitro* experiments, DZR was added to the cell culture medium at a concentration of 100, 200, or 400 μM before irradiation per the protocol described in a previous study [41].

### Rat heart irradiation and DZR administration

**Figure 5:**
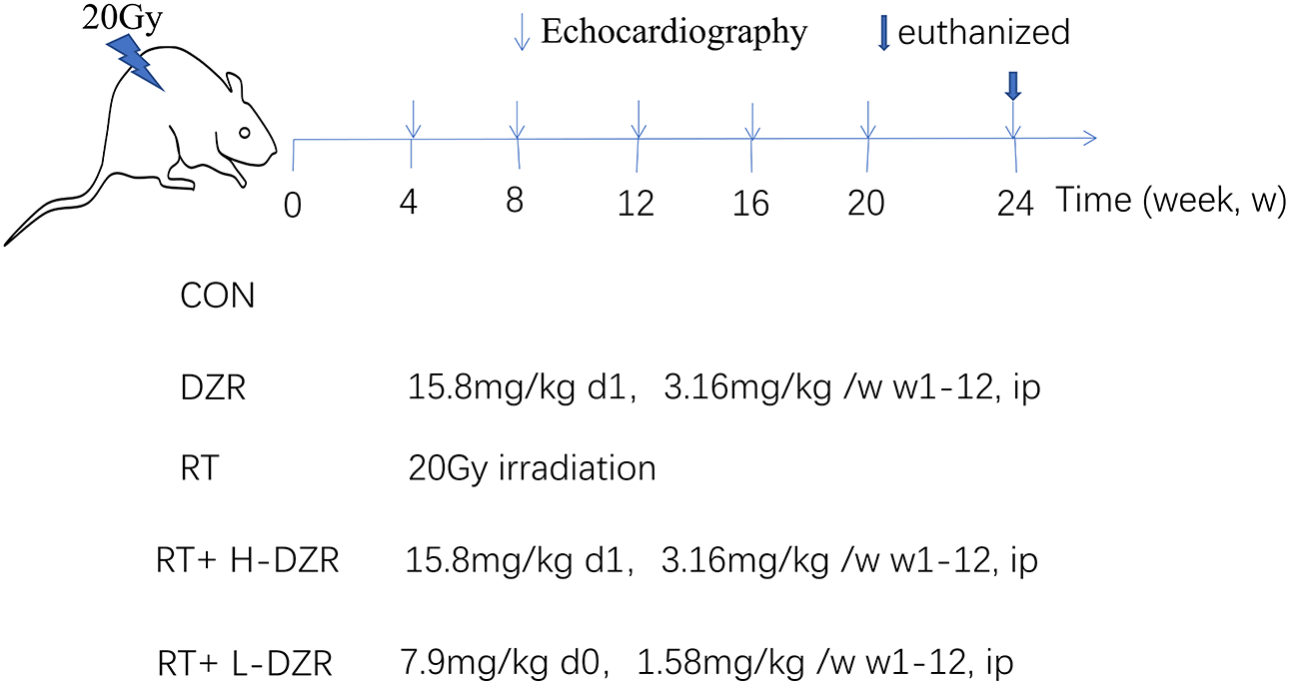
**Dexrazoxane treatment.** A single 20 Gy dose was administered to the entire rat heart to simulate RIHD; DZR treatment was administered for 12 weeks post-irradiation. Three randomly selected rats from each group were monitored using high-frequency echocardiography at 4, 8, 12, 16, 20, and 24 weeks post irradiation. All rats were euthanized at 24 weeks; serum and heart tissue samples were collected.

Sixty-five male Sprague-Dawley rats aged 8–10 weeks were obtained from the Experimental Animal Center of Hubei Province and housed in our pathogen-free animal facility with free access to food and water. After one week of acclimatization, they were randomly divided into five groups: control (CON, n=10), dexrazoxane (DZR, n=10), radiation (RT, n=15), radiation with high-dose DZR (a single i.p. dose of 15.6 mg/100 g immediately after irradiation followed by 3.12 mg/100 g i.p. per week until 12 weeks after irradiation, RT+H-DZR, n=15), and radiation with low-dose DZR (a single i.p. dose of 7.8 mg/100 g immediately after irradiation followed by 1.56 mg/100 g per week i.p. until 12 weeks after irradiation, RT+ L-DZR, n=15) (Figure 5).

Rats in the irradiated groups were anesthetized using intraperitoneal chloral hydrate (7%). The whole heart was irradiated with a single dose of 20 Gy using a linear accelerator. Irradiation was performed according to previously published protocols [42, 43] and based on post-anatomical heart size measurements. A medical linear accelerator (Elekta Precise) was used to irradiate one rat at a time. The rat’s limbs were fixed on the plate in the supine position, the center of the irradiation field was aligned with the geometric center of the heart, and a source distance of 100 cm, 180° field, irradiation field size of 2.0 cm×2.0 cm, dose rate of 500 cGy/min, and total dose of 20Gy (6MV X-ray) per administration were used.

All procedures in this study were approved by the Institutional Animal Care and Use Committee of Tongji Hospital affiliated with the Tongji Medical College of Huazhong University of Science and Technology.

### Echocardiography

High frequency (30 MHz) echocardiography (Vevo 2100, VisualSonics Inc, Toronto, Canada) was performed once every month for up to 6 months after irradiation to assess left ventricular function. After shaving their chest hair, rats were initially anesthetized with a 5% isoflurane-O_2_ mixture in an induction chamber, and 2% isoflurane-O_2_ was then administered during ultrasound procedures while dynamic electrocardiograms were recorded using a gas anesthesia system (isoflurane vaporizer; Ohio Medical Products). M-mode echocardiograms were recorded and analyzed.

### Tissue collection and histology

All rats were sacrificed 24 weeks after irradiation. Their hearts were isolated and a tissue sample from each heart was used for TEM. Other tissue samples were partially embedded in paraffin and stained with hematoxylin-eosin and Masson’s trichrome [44]. The remainder of the cardiac tissue was used for transcriptome sequencing, TUNEL staining, and ROS detection.

For TEM, a 1 mm^2^ piece of cardiac tissue was fixed with 2.5% glutaraldehyde and washed with phosphate buffer several times. The tissue was sequentially fixed in 1% osmium acid for one hour and then dehydrated using a series of acetone treatments at room temperature. The sample was then embedded in Epon 618 and cut into ultrathin sections using an ultramicrotome after staining with uranyl acetate and lead citrate. These sections were studied using a transmission electron microscope (HT7700, Hitachi, Tokyo, Japan).

### TUNEL assay

To identify myocardial apoptosis, a TUNEL assay was performed on cardiomyocytes using a TUNEL kit (11684817910, Roche, Swiss) and according to the manufacturer’s protocol. TUNEL-positive cardiomyocytes were those that contained both TUNEL staining and DAPI-positive nuclei in heart tissue. In each TUNEL-stained section, five fields were randomly chosen and analyzed using fluorescence microscopy. All slides were individually analyzed by two individual observers in a blinded manner.

### Measurement of ROS production

To measure ROS levels *in situ* using DHE staining, rat heart tissues (n=5 per group) and tumor tissues from nude mice (n=5 per group) were cast into 10 μm frozen sections (LeicaCM1850; Leica Microsystems GmbH). Following the manufacturer’s protocols, these sections were incubated with 5 mM dihydroethidium (KGAF019, Nanjing KeyGen Biotech Co., Ltd., Nanjing, China) for 30 minutes at 37° C. Fluorescence images were obtained using a Carl Zeiss Axioscope A1 microscope. For DHE staining, five fields per sample were randomly chosen and analyzed. DHE fluorescence intensity was quantified using Image Pro Plus (version 1.4.3.67, NIH).

### Transcriptome sequencing and data analysis

Total RNA was isolated from cardiac tissues of CON and RT group rats (n=3 per group) to construct cDNA libraries which were then sequenced using the Illumina HiSeq^TM^ 2000 platform (Illumina, San Diego, CA, USA). A *p*-value<0.05 and |log_2_(foldchange)<1| were used to identify differentially expressed genes (DEGs) between the two groups. Relative abundances were analyzed using a heat map. Kyoto Encyclopedia of Genes and Genomes (KEGG) analysis was used to identify biological functions and molecular interaction networks associated with the DEGs.

### Tumor formation experiment

Male nude BALB/C mice (4 weeks old, Experimental Animal Center of Hubei Province, Wuhan, China) were housed in the animal facility of our hospital and allowed to adapt to the environment for one week prior to treatment. H292 cells were digested and collected in a serum-free medium. The suspension was subcutaneously injected into the left axilla of each nude mouse (1×10^6^ cells suspended in 0.2 mL medium per mouse). Tumor size was measured every three days using a Vernier Caliper once the tumors were palpable, and the weight of each mouse was recorded at this time. The tumor volume (mm^3^) was equal to length × width × width × 0.5 [45].

After xenograft tumors reached a mean size of 20–50 mm^3^, the mice were randomly divided into 4 groups (n=7 per group): CON, DZR, RT, and RT+DZR. In the experimental groups, tumors were irradiated with a single 10 Gy dose using a linear accelerator, and DZR was delivered as a single dose of 15.6 mg/100 g immediately after irradiation followed by 3.12 mg/100 g per week until the mice were euthanized. All mice in a group were euthanized when tumor length of most mice in the group exceeded 15 mm. The tumors were collected for immunohistochemical analysis.

### Western blot analysis

Total cellular or tissue protein was extracted using RIPA lysis buffer. Proteins were separated using electrophoresis, transferred onto a polyvinylidene fluoride membrane, and separately incubated with the following primary antibodies: GAPDH (AC002, 1:5000, Abclonal, Wuhan, China), Caspase-3 (9662s, 1:1000, Cell Signaling Technology), Cleaved Caspase-3 (9664T, 1:1000, Cell Signaling Technology), Bcl-2 (BA0412, 1:200, Boster, Wuhan, China), Bax (5023T, 1:1000, Cell Signaling Technology). The membranes were washed and incubated with the corresponding secondary antibody (HRP-goat anti-rabbit IgG or goat anti-mouse antibody, 1:10000) for 1 h at room temperature. Proteins were visualized using enhanced chemiluminescence and the G: BOX Chemi X system (Syngene) per the manufacturer’s guidelines.

### Immunohistochemical analysis

For immunohistochemistry, paraffin-embedded heart sections were deparaffinized, rehydrated, and subjected to antigen retrieval. Next, samples were incubated with 5% bovine serum albumin at 4° C overnight with the primary antibody (NF-κB p65, 1:200, BIOSS, bs-0465R). The sections were then washed with TBST, incubated with peroxidase polymer-conjugated secondary antibody (1:5000) and DAB (both from Aspen Biological), and counterstained with hematoxylin. Three fields in each slice were selected and observed under 400× magnification. Brownish yellow nuclear staining was indicative of p65-positive cells. Total cell numbers and p65-positive cell numbers were counted to calculate the p65-positive cell ratio.

### Cardiac troponin I assay

Blood samples were collected from rats at week 24 after RT from the heart apex at the moment of sacrifice, stored on ice, and subsequently centrifuged at 3000 ×g at 4° C for 15 min to obtain serum samples. Serum cTnI levels were assessed using a cTnI Assay Kit (ELK1552, ELK Biotechnology, Wuhan, China).

### Statistical analysis

Results are expressed as the mean ± standard error of the mean (SEM). All data were statistically analyzed using a one-way analysis of variance with Bonferroni's correction for multiple groups or Student’s t-test for two groups using GraphPad Prism 8.0 (GraphPad Software, CA, USA). The overall survival analysis was performed using the Kaplan-Meier method and evaluated using the log-rank test. *p*<0.05 was considered statistically significant.
